# Embolization of renal tumor bone metastasis: case report

**DOI:** 10.1590/1677-5449.210005

**Published:** 2021-06-21

**Authors:** Felipe Soares Ribeiro, Salim Abdon Haber Jeha, José Victor Figueiredo dos Santos, Ananda Vitória Suzuki Barros Damasceno, Tereza Maria Meireles Fernandes da Silva, Fernando Brasil do Couto, Humberto Balbi Reale

**Affiliations:** 1 Hospital Porto Dias, Belém, PA, Brasil.; 2 Universidade Federal do Pará – UFPA, Belém, PA, Brasil.; 3 Faculdade Metropolitana da Amazônia – UNIFAMAZ, Belém, PA, Brasil.; 4 Universidade do Estado do Pará – UEPA, Belém, PA, Brasil.

**Keywords:** renal cell carcinoma, arterial embolization, bone metastasis, carcinoma de células renais, embolização arterial, metástase óssea

## Abstract

Primary or secondary bone tumors can manifest in different ways, from simple bone pain to possible pathological fractures. Hypervascularized tumors are of greatest concern, with increased incidence of complications. Preoperative embolization of the bone tumor is an effective measure for reducing blood loss during open surgery to excise the tumor. With appropriate experience, the risks of the procedure are minimal and final outcomes are highly satisfactory. The purpose of this paper is to describe the case of a 43-year-old male patient with a metastatic renal cell tumor in the left proximal femur (seen on lower limb computed tomography) who underwent selective preoperative embolization. The procedure resulted in a remarkable absence of bleeding and successful response to subsequent onco-orthopedic surgery.

## Resumo

Os tumores ósseos, primários ou secundários, podem se manifestar de várias formas, desde dor óssea até fraturas patológicas. A preocupação maior repousa sobre tumores hipervascularizados, com maior incidência de complicações. A embolização pré-operatória do tumor ósseo é uma medida eficaz para reduzir a perda sanguínea durante a exérese tumoral em cirurgia aberta. Com uma experiência apropriada, os riscos do procedimento são mínimos, com resultados finais bastante satisfatórios. O presente artigo tem por finalidade descrever o caso de um paciente do sexo masculino, de 43 anos, com tumor metastático de células renais em fêmur proximal esquerdo (visualizado por angiotomografia de membro inferior), submetido à embolização arterial seletiva pré-operatória. O procedimento resultou em ausência de sangramento e ótima resposta à cirurgia onco-ortopédica realizada.

## INTRODUCTION

Kidney cancer is the twelfth most common type of malignancy worldwide, with incidence in males twice the rate in females.^1^^,^^2^ Metastases are frequent in renal neoplasms and the bone is the second most common site, with an incidence of 30%.^3^

Locoregional treatments have emerged as an important element for symptom control, improving therapeutic results and offering quality of life to these patients.^4^

Arterial embolization is defined as a locoregional treatment to selectively devascularize hypervascularized tumors, slowing their growth without affecting adjacent organs.^5^ It also delivers a more positive prognosis, enabling pain reduction, reducing the risk of spontaneous bleeding, and decreasing the likelihood of bleeding during orthopedic oncologic surgery.^6^

This study aims to report the results of selective arterial embolization performed in a patient with a bone metastasis from renal carcinoma, attesting to the functionality and benefit of this procedure as an important element in satisfactory cancer treatment outcomes.

The protocol was approved by the institution Ethics Committee (Decision number 4.582.420).

## CASE REPORT

The patient was a 43-year-old Brazilian man who works as a store salesperson and had a medical history of nephrectomy and surgical resection of a metastatic spine lesion about 12 months earlier. After these procedures, he was diagnosed with metastatic lesions in the retroperitoneum, bones, and lung. He was admitted to the emergency room of our service complaining of severe pain in his left thigh.

**Figure 1:**
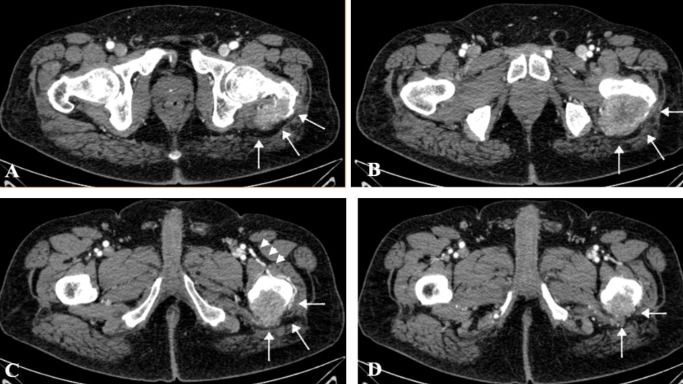
Angio-CT axial slice showing renal cell carcinoma metastasis at the femoral neck. (**A**) Tumor apex at the femoral neck (white arrows); (**B**) Proximal third of Bone Tumor (white arrows); (**C**) Distal third of Bone Tumor (white arrow) and arterial supply of the deep femoral artery (white arrowhead); (**D**) Bottom of the Bone Tumor.

Upon initial physical examination, he was in regular condition, without fever, and hemodynamically stable. Computed tomography was performed, revealing a lytic lesion on the left proximal femur neck, MIRELS classification 11,^7^ with a high risk of a pathological fracture (Figure 1). The diagnostic hypothesis was metastatic bone lesion of the proximal femur derived from a renal cell carcinoma.

**Figure 2:**
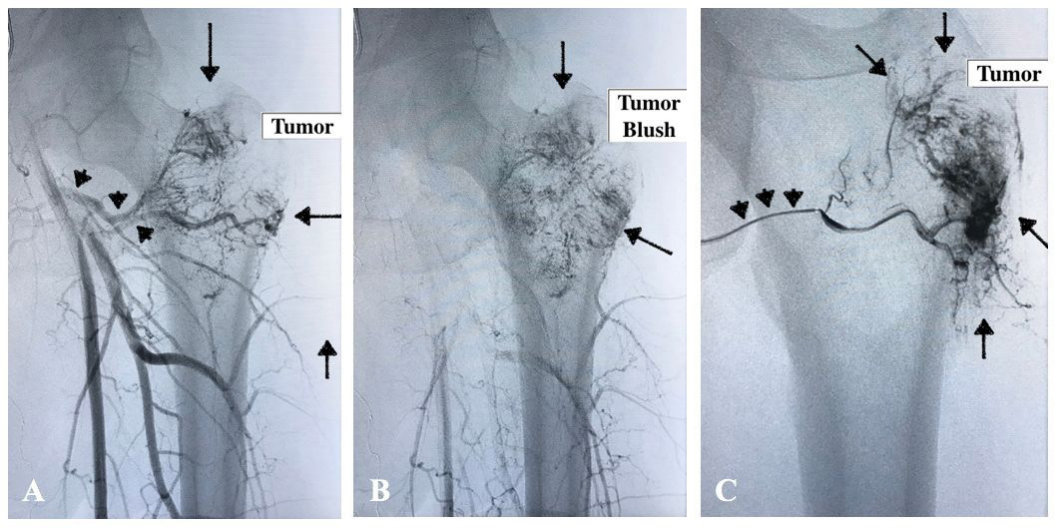
Left limb arteriography. **A)** Arterial supply to the tumor by deep femoral artery branches (black arrowheads) and initial tumor blush at the femoral neck (black arrows); **B)** Tumor blush at the left femoral neck (black arrows); **C)** Superselective arterial catheterization (black arrowheads) showing tumor blush (black arrows)

**Figure 3:**
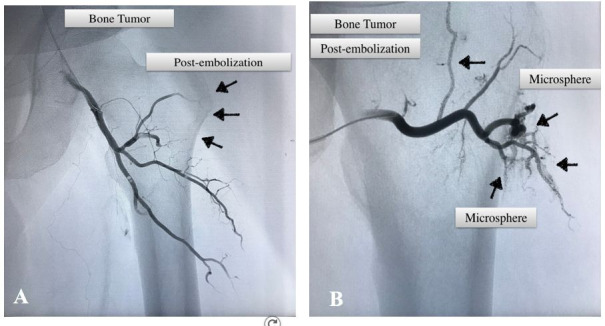
Post-embolization left limb arteriography. **A)** Absence of tumor blush at femur neck (black arrows); **B)** Microspheres inside arterial branches of the tumor (black arrows).

The patient was referred to the vascular surgery team for evaluation and preoperative embolization was indicated. Subsequently, the oncologic orthopedics team performed an open resection of the proximal femur and replacement with prosthesis. For the embolization, the patient was placed in the horizontal supine position under spinal anesthesia. The right common femoral artery was punctured with a 5-French introducer sheath. A pigtail catheter and a 0.035 hydrophilic guidewire were used to cross the contralateral iliac territory and catheterize the left common femoral artery. Arteriography was performed from multiple incidences, showing a tumor blush at the proximal femur neck (Figure 2) with blood supply from deep femoral branches (lateral femoral circumflex artery and smaller branches). The deep femoral branches were catheterized with a Vert catheter and the smaller tumoral branches with a Progreat® microcatheter (Terumo Corporation, Shibuya, Tokyo, Japan). Subsequently, embolization was performed with Embosphere® 300-500 μm microspheres (Merit Medical, Utah, USA), obtaining complete devascularization on the final arteriography (absence of tumor blush) (Figure 3).

**Figure 4:**
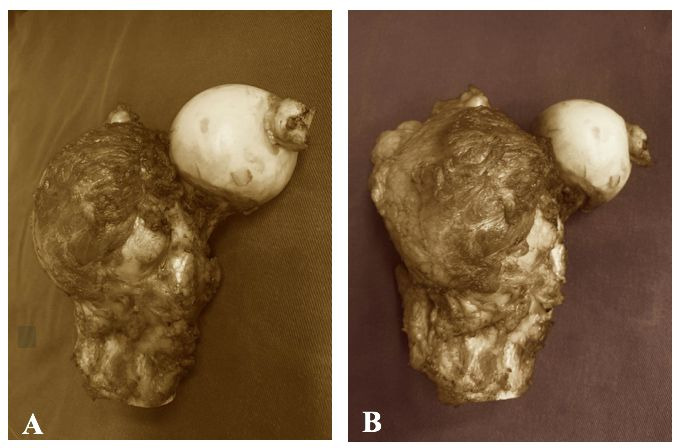
**A** and **B)** Bone tumor – resected renal cell carcinoma metastasis.

After 48 hours, the patient underwent an orthopedic procedure under spinal anesthesia. A 20cm lateral incision was made in the proximal left thigh, followed by dissection until complete tumor visualization was achieved. An 11cm section of the proximal left femur was removed along with the tumor mass (Figure 4). There were no intraoperative complications, bleeding, or need for blood transfusion. The surgical specimen was sent to the pathology department and was later confirmed to be a bone metastasis from a renal cell carcinoma.

The patient maintained follow-up with the oncology and orthopedics teams. He underwent a chemotherapy regimen for renal cell carcinoma. Positive results were obtained. There were significant reductions in pain score and need for analgesics and improvement in quality of life.

## DISCUSSION

Renal cell carcinomas have a high incidence of bone metastasis. They are one of the metastatic diseases with most embolization reports in the literature.^8^ These bone tumors are hypervascularized, especially when they originate from renal cell carcinomas, and they are a cause for considerable concern with regard to bleeding during open surgical procedures.^9^^,^^10^

In the mid-1970s, superselective and selective embolization was used to reduce perioperative bleeding in a case series of bone tumor embolizations.^11^ Since then, selective arterial embolization has emerged as an isolated procedure or as part of a broader therapeutic set, bringing benefits such as pain relief (decompression of the periosteum and reduction of neurological symptoms) and improving these patients’ quality of life.^12^^,^^13^ Therefore, considering the excellent pain control results, some authors believe it to be a first-line palliative therapy.^14^^,^^15^

According to Pazionis et al., not performing preoperative embolization may result in massive bleeding with an increased risk of patient morbidity. Furthermore, in patients at high cardiological risk, severe bleeding can also increase mortality and embolization may be essential for this reason.^16^^,^^17^

Since its advent, several techniques and materials have been tested to improve the embolization procedure. The case series by Feldman et al. used Gelfoam® for embolizations, but this substance has only a temporary occlusive effect, with recanalization of the vessel occurring in a few weeks. Other embolic agents have been developed. Currently, polyvinyl alcohol particles, N-butyl-cyanoacrylate, and trisacryl microspheres are available.^4^ In this study, the microsphere embolization technique was used because it is applicable to vessels of smaller caliber, in addition to the more uniform and homogeneous size.

A study compared a group of patients comprising 27 kidney cancer patients and 12 thyroid cancer patients who underwent selective bone tumor embolization prior to surgery to a group of 41 patients who did not. This comparative study demonstrated reduced blood transfusion requirements (2.15 versus 3.56 U; p = 0.020), diminished blood loss (0.90 versus 1.77 L; p = 0.002), and shorter operation time (3.13 versus 3.91 hours; p < 0.001) for the group treated with embolization, demonstrating the functionality reported by the literature and the significance of this procedure.^16^

In patients with indications for tumor resection, one relevant factor in post-embolization surgical success is the period between the two procedures, considering that after a few days there is a progressive increase in local neovascularization and, therefore, the bleeding risk returns.^17^ Current literature recommends that the open surgery be carried out at most three days after the embolization.^17^ After that, neoangiogenesis occurs and there is a new cumulative risk of bleeding. The patient in this case report was successfully subjected to open left femur resection with prosthetic replacement 48 hours post-embolization.

Although rare, embolization involves some risks inherent to the procedure, with emphasis on arterial dissection; accidental embolization of adjacent vessels; muscle necrosis; transient paresthesia in the lower limbs (most common); and contrast nephropathy. However, the incidence of serious complications, such as acute ischemia, is lower than 1%. The procedure is therefore considered to be quite safe and has a low morbidity rate when performed by an experienced team.^5^^,^^16^

Currently, only one case series demonstrating the experience with the bone tumor embolization technique is available in the literature. It is therefore necessary to conduct prospective studies to evaluate not only short-term results, but also the late postoperative period and the impact on the survival of these patients over an extended follow-up.

Selective and superselective preoperative embolization of renal cell carcinoma metastasis plays an important role among oncological treatment options. It offers patients greater autonomy, better pain control, independence, and improved quality of life.

## CONCLUSION

Arterial embolization with microspheres is a safe and effective preoperative treatment for patients with bone metastasis from renal cell carcinomas, with significant clinical benefits especially regarding pain relief, low complications rates, and a significant reduction in the risk of intraoperative hemorrhage during the oncological orthopedic procedure (tumor resection and replacement with prosthesis).
